# Effectiveness of regular oat β-glucan–enriched bread compared with whole-grain wheat bread on long-term glycemic control in adults at risk of type 2 diabetes: a randomized controlled trial

**DOI:** 10.1016/j.ajcnut.2025.06.018

**Published:** 2025-06-24

**Authors:** Therese Hjorth, Alena Schadow, Ingrid Revheim, Ulrike Spielau, Klara Meyer, Anne Rieder, Paula Varela, Simon Ballance, Antje Koerner, Rikard Landberg, Anette E Buyken, Jutta Dierkes, Hanne Rosendahl-Riise

**Affiliations:** 1 Division of Food and Nutrition Science, Department of Life Sciences, Chalmers University of Technology, Gothenburg, Sweden; 2 Faculty of Natural Sciences, Institute of Nutrition, Consumption and Health, Paderborn University, Paderborn, Germany; 3 Department of Clinical Medicine, Centre for Nutrition, University of Bergen, Bergen, Norway; 4 Medical Faculty, Center for Pediatric Research, Hospital for Children and Adolescents, Leipzig University, Leipzig, Germany; 5 Nofima AS, Norwegian Institute of Food, Fisheries and Aquaculture Research, Ås, Norway

**Keywords:** β-glucans, type 2 diabetes, glycated hemoglobin (HbA1c), glycemic control, randomized controlled trial, effectiveness trials

## Abstract

**Background:**

A high intake of whole grains is associated with reduced risk of type 2 diabetes and cardiovascular disease, and soluble fiber from oats and barley, that is, β-glucans, has been shown to lower blood cholesterol and postprandial glycaemia. Despite such data and the European Food Safety Authority health claims supporting β-glucan–induced reductions in glucose and cholesterol, effectiveness in real-life settings among individuals at elevated risk of developing type 2 diabetes remains unclear.

**Objectives:**

This study aims to assess the long-term effectiveness of daily consumption of β-glucan–enriched bread, compared with whole-grain wheat bread, on glycated hemoglobin (HbA1c) and glycemic control in adults at risk of type 2 diabetes.

**Methods:**

A 16-wk randomized, double-blind dietary intervention was conducted in 194 adults [58 ± 8 y; BMI: 32 ± 5 kg/m^2^; HbA1c 5.6% ± 0.3% (38 ± 3 mmol/mol); LDL cholesterol 3.6 ± 1.0 mmol/L] across sites in Germany, Norway, and Sweden. Participants consumed ≥3 slices/d of either β-glucan–enriched bread (6 g β-glucan/d) or control bread, 6 d/wk.

**Results:**

After 16 wk, there was no significant between-group difference in HbA1c [Δ = −0.01%, 95% confidence interval (CI): −0.03, 0.06; *P* = 0.49]. Similarly, no differences were observed in fasting glucose (Δ = −0.02 mmol/L; 95% CI: −0.11, 0.14), insulin (Δ = −0.76 pmol/L; 95% CI: −0.99, 2.5), or LDL cholesterol (Δ = −0.11 mmol/L; 95% CI: −0.27, 0.05) (all *P* > 0.05).

**Conclusions:**

Contrary to expectations from efficacy studies, this effectiveness trial does not support the metabolic benefits of oat-derived β-glucan–enriched bread under real-life conditions. A simple bread replacement may not be sufficient to improve glucose homeostasis in individuals at risk of type 2 diabetes.

This trial was registered with clinicaltrials.gov as NCT04994327.

## Introduction

A higher dietary fiber intake is associated with a lower risk of developing type 2 diabetes and cardiovascular diseases and beneficial effects on metabolic risk factors such as body weight, lipid profile, and blood pressure [[1], [2], [3]]. In European diets, cereal foods are a main source of dietary fiber [4,5], and dietary guidelines emphasize the preferred consumption of whole grains [6], but there is no distinction on the whole grain type [5]. Whole grains rich in viscosity-forming β-glucans from oats and barley may be particularly beneficial for cardiometabolic disease prevention, as supported by data from studies on acute postprandial glucose response and blood cholesterol levels [7,8]. β-glucans are fiber molecules with mixed-linkage β-glucan bond patterns, resulting in varying molecular weight and water solubility, which influence their metabolic effects [9].

On the basis of acute and short-term intervention studies, that is, efficacy studies, European Food Safety Authority (EFSA) has approved health claims of the beneficial effects of β-glucans on postprandial glucose and blood lipid concentrations [10]. According to EFSA, 4 g of oat or barley β-glucan per 30 g of available carbohydrates will reduce postprandial glucose response, and 3 g of β-glucan reduce blood cholesterol among individuals with slightly elevated cholesterol levels [10]. A systematic review and meta-analysis by Chen et al. [11] that included 8 trials with 407 participants with type 2 diabetes, reported a glycated hemoglobin (HbA1c) reduction of 0.47% (5.1 mmol/mol) and a fasting glucose decrease of 0.75 mmol/L at a median β-glucan intake of 3.25 g/d over 4.5 wk. However, the study noted large variations in β-glucan characteristics and study designs, leading to low-quality evidence for HbA1c effects. Similarly, a meta-analysis by Shen et al. [12] found significant reductions in fasting glucose (−0.52 mmol/L) and HbA1c (−0.21%, −2.3 mmol/mol) at median β-glucan intakes of 2.5–3.5 g/d over 3–8 wk.

Although controlled intervention studies and EFSA-approved health claims demonstrate the effect of β-glucans under ideal conditions, understanding the effectiveness of β-glucans under real-life conditions is crucial for public health recommendations. To our knowledge, no study has yet evaluated the effectiveness of incorporating oat β-glucan into a habitual diet under real-life conditions without controlling other dietary factors in individuals at risk of type 2 diabetes. Modifying the habitual diet, such as substituting staple foods with healthier options, represents a practical strategy to prevent or mitigate progression toward type 2 diabetes [13].

The aim of the study was to investigate the effectiveness of consuming oat β-glucan–enriched bread as part of a habitual diet to improve glycemic control in adults at high risk of type 2 diabetes, compared with whole-grain control bread. HbA1c was the primary outcome, with fasting glucose and insulin, lipid profile, insulin sensitivity indices, body composition, and hepatic steatosis markers as secondary outcomes. Furthermore, consumer acceptance of the breads was evaluated.

## Methods

The CarbHealth trial is a multicenter, double-blind, parallel, randomized, controlled 16-wk dietary intervention trial in participants with HbA1c concentrations in the range 35–50 mmol/mol at screening. The study protocol has been published elsewhere, and the outline of the study is described in brief [14]. The recruitment started in July 2021, and the trial was finalized in September 2023. The study was conducted at 4 universities in 3 countries: *1*) University of Bergen, Bergen, Norway, *2*) Chalmers University of Technology, Gothenburg, Sweden, *3*) Paderborn University, Paderborn, Germany, and *4*) Leipzig University, Leipzig, Germany.

The study was conducted according to the Declaration of Helsinki and in accordance with the guidelines for Good Clinical Practice.

### Institutional review board statement

The study protocol was approved by the respective ethic authorities [Swedish Ethic Review Authority (protocol DNR 2021-02584), the ethical committee of Paderborn University, Paderborn (approved 13 July, 2021), the ethical committee of the medical faculty of the University of Leipzig, Leipzig (316/21-ek), and regional committees for Medical and Health Research Ethics, Norway (REC Nord, ref. 106931)].

### Recruitment

Participants were recruited via leaflets, press releases, newspaper articles, ads on social media, radio, and blackboard flyers. All participants screened for eligibility were provided with written information about the study before the first study visit. All participants gave written consent to study participation.

### Eligibility criteria and randomization procedures

Regular bread consumers aged 40–70 y, with BMI ≥27m^2^, and HbA1c 5.4%–6.7% (35–50 mmol/mol) were eligible for inclusion. The HbA1c range was selected to include individuals at increased risk of type 2 diabetes, including those with slightly lower or higher HbA1c values than the standard prediabetes range of 5.7%–6.4% (39–47 mmol/mol), to reflect a broader real-world risk spectrum, than only clinically defined individuals with prediabetes. Exclusion criteria were: diagnosed type 1 diabetes mellitus or pharmacologically treated type 2 diabetes, fasting blood glucose >7.0 mmol/L, nonfasting blood glucose >11.1 mmol/L, urine glucose >180 mg/dL or urine protein excretion, food allergies preventing consumption of study breads, pregnancy, lactation, or planning a pregnancy within the intervention period, systolic blood pressure ≥160 mmHg and/or diastolic blood pressure ≥100 mmHg, history of gastrointestinal symptoms, myocardial infarction, heart failure, stroke, heart attack, or cancer within 3 y before screening, and history of alcohol abuse. Furthermore, the use of antidiabetic agents at screening or initiation of medication to treat diabetes during the trial was reason for exclusion.

Participants were randomly assigned into 1 of the 2 treatment groups (1:1 allocation) using block randomization with random block lengths stratified by sex and study center [14].

### Intervention

During the 16-wk intervention period, participants were instructed to replace their habitually consumed bread with 3–6 slices of presliced intervention oat bread or control bread per day, on ≥6 d/wk. The participants were free to consume the bread whenever they wanted during the day. They were also instructed to maintain their habitual diets and level of physical activity. The study breads were specifically developed and produced for the trial by the Norwegian Institute for Food, Fisheries and Aquaculture Research (NOFIMA) and were provided frozen for free to the participants.

### Study breads

The daily portion of 3 slices of oat bread provided 286 kcal total energy, 16.6 g dietary fiber, and 6.0 g of β-glucan. Three slices of the control whole-grain wheat bread provided 244 kcal total energy, 5.0 g dietary fiber, and 0.02 g of β-glucan. The two breads were matched in starch and fat content on a slice basis. The weight-average molecular weight of the β-glucan in the oat bread was ∼1000 kDa and was measured, as described by Mæhre et al. [15]. To confirm that the analyzed material was β-glucan, the sample was treated with lichenase according to the procedure described by Ballance et al. [16]. A full list of ingredients and macronutrient composition has previously been published [14].

### Blinding

This was a double-blind trial. Participants were blinded to their allocated groups, and the bread was delivered in similar packaging. Because of a slight difference in bread color, participants were not allowed to directly compare the breads during clinic visits. Research staff conducting clinical examinations and sample collection was unaware of group allocation. Additionally, group allocation remained blinded during statistical analyses.

### Clinical assessments and blood collection

Clinical visits were conducted at baseline, mid-intervention (week 8), and post-intervention (week 16), with primary outcomes assessed at baseline and week 16. Participants arrived in the morning after a 10-h overnight fast and refrained from alcohol, tobacco use, and vigorous physical activity for ≥12 h before each visit. Clinical visits included the collection of fasting blood, fecal, and urine samples; anthropometric measurements; and blood pressure assessments. Full description of methodological details is published elsewhere [14].

Blood was drawn from an antecubital vein, processed, and frozen at −80 °C until analysis. HbA1c was measured in EDTA whole blood by HPLC, and C-reactive protein (CRP) was measured in serum using standardized enzymatic methods in a certified clinical laboratory (NS-EN ISO 15189, Haukeland University Hospital). Liver enzymes and plasma lipids were analyzed using standard methods on a Cobas c702 autoanalyzer (Roche Diagnostics). Liver enzymes were measured photometrically according to the International Federation of Clinical Chemistry method. Serum triglycerides and total cholesterol were assessed using enzymatic colorimetric methods. LDL cholesterol was measured photometrically, and HDL cholesterol was measured using a homogeneous enzymatic colorimetric method. Blood samples were analyzed in batches at the Department of Medical Biochemistry and Pharmacology at Haukeland University Hospital, Bergen, Norway, with a maximum storage time for HbA1c samples of 8 wk.

Blood pressure was measured 3 times at 1-min intervals using a digital monitor (Omron HEM-907) after the participant had rested for 5 min in an upright, seated position. The mean of the three measurements was used in the analysis.

### Anthropometric measurements

Body weight was measured to the nearest 0.1 kg using calibrated scales, with participants wearing light clothing and no shoes. Height was measured without shoes using a stadiometer. Waist circumference was measured twice according to WHO standards, and the average was used for analysis. Body composition (fat mass and fat-free mass) was assessed using bioelectrical impedance analysis (Seca mBCA 515) in Bergen, Paderborn, and Leipzig, and by dual-energy X-ray absorptiometry (DXA; iDXA, GE Healthcare) in Gothenburg. All measurements were performed following standardized procedures.

### Dietary assessment, bread consumption, and consumer evaluation

Dietary assessment was based on 6 repeated 24-h recalls at 3 time points: weeks 0–2, 7–9, and 15–16, using country-specific food data. In Bergen, Paderborn, and Leipzig, "MyFood24" was used, whereas in Gothenburg, recalls were conducted on-site and by phone using Swedish food images and household measures. Energy and macronutrient averages were calculated from the first, middle, and last 2 recalls. Full dietary assessment details are available elsewhere [14]. Participants recorded bread consumption by a precoded journal ticking off numbers of slices consumed each day. Participants evaluated the bread on day 1 and week 8 using a questionnaire described in Hjorth et al. [14], rating hunger, acceptability, expected satiation, and answering 2 consumption questions plus a check-all-that-apply question.

### Metabolic indices

The HOMA-IR was calculated using the approach developed by Matthews et al. [17]. The Quantitative Insulin Sensitivity Index (QUICKI) was derived from the method proposed by Katz et al. [18]. For the Hepatic Steatosis Index, the calculation followed the method of Lee et al. [19], whereas the Fatty Liver Index (FLI) was determined using the formula established by Bedogni et al. [20]. For analyses involving CRP, participants with CRP values > 4.1 mg/L were excluded, as elevated levels may indicate acute or chronic inflammation that could confound interpretation of this outcome [21].

### Lifestyle and sociodemographic characteristics

Physical activity was assessed using the International Physical Activity Questionnaire (IPAQ), and was categorized into 3 levels: high, medium, and low [22]. Education level was categorized into 3 groups: low (<11 y of schooling), medium (high school and adult education), and high (including university degrees at the bachelor, master, and PhD levels). Self-reported ethnicity was recorded during the baseline visit and categorized as Caucasian, Asian, African, or other/prefer not to answer.

### Statistical analysis and sample size estimates

The statistical power calculation was based on detecting a difference in HbA1c between the two groups at the end of the intervention. We expected the starting HbA1c to be ∼41 mmol/mol with an SD of 6 mmol/mol and estimated a reduction to 38 mmol/mol in the oat group, with small or no change in the control group. To detect a difference between groups in HbA1c of 3 mmol/mol with an SD of 6 mmol/mol and a power of 90% at a significance level of 0.05, we initially needed ∼163 participants. After accounting for a 45% dropout rate, the final required sample size was 250 participants, with 125 in each treatment group. Assumptions and estimates for the power calculation were taken from previous dietary intervention studies [23,24] and the calculation was made with a paired *t* test using the power *t* test from R. The effect size was hypothesized to be moderate as this is a single-food intervention study in a group of individuals at slightly elevated diabetes risk. Details are given elsewhere [14].

The primary analysis was an intention-to-treat (ITT) analysis where the ITT population consisted of all subjects who were randomized and attended the baseline visit. Missing data were imputed by multiple regression imputation methods. Missing values were predicted based on age, waist circumference, BMI, body fat mass, and sex. The complete-case population (CC) consisted of all subjects with complete data on the outcome of interest without any major protocol deviations. The CC analyses are presented in Supplemental Tables 1–5. Differences between the groups were analyzed by generalized linear model where the difference between baseline and week 16 was the dependent variable, treatment group as independent variable, and baseline value and center were included as covariates. An ad hoc analysis comparing the highest and lowest consumer groups (based on a median split) was conducted. A sensitivity analysis was conducted to assess whether results for blood lipid outcomes were influenced by lipid-lowering medication use. Analyses were repeated with adjustment for lipid-lowering treatment (yes/no) as a covariate.

The main statistical analyses were carried out using R Studio (version 4.4.0, May 2024, for Windows 10) [25]; estimates were derived using the car-package [26] and visualization was made with the ggplot2 package [27]. For consumer acceptance, satiety, and satiation, only the CC analysis was performed. Minitab v21 [28] was used employing a general linear model with 3 fixed factors: day 1/week 8, bread type, and country. Posthoc testing was performed using the Tukey test. Check-all-that-apply data were analyzed using Cochran and McNemar tests in EyeOpenR [29].

Data are expressed as mean ± SD (M ± SD) if not otherwise stated. *P* values are presented with corresponding 95% confidence interval. An α value of 0.05 was set as the significance level.

## Results

**FIGURE 1:**
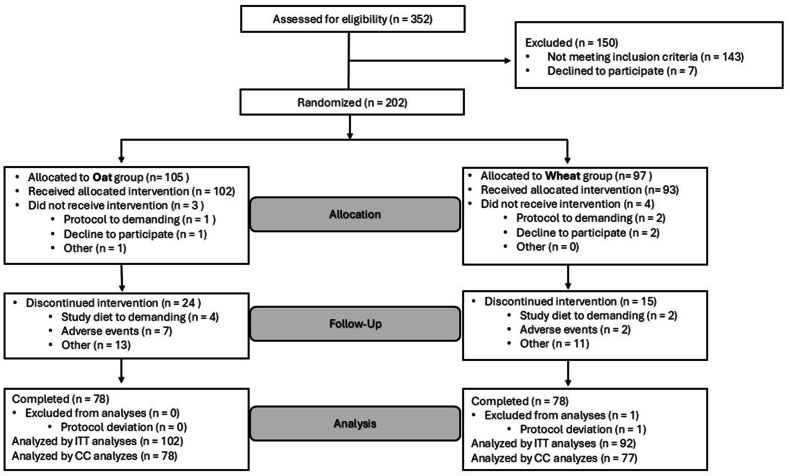
CONSORT flow diagram. Flow of participants through the CarbHealth study, including enrollment, randomization, allocation to intervention or control groups, follow-up, and analysis. CC, complete case; ITT, intention-to-treat.

During the recruitment phase, 352 participants were screened for eligibility and 202 were enrolled in the study. Of the initially recruited 202 participants, 7 individuals (oat *n* = 2; control *n* = 5) dropped out before starting the trial, and 38 individuals (oat *n* = 23; control *n* = 15) dropped out during the intervention (Figure 1). One individual in the control group was excluded from analyses due to protocol deviations. Seven individuals in the oat group and 2 individuals in the control group dropped out because of adverse events such as stomach problems. No serious adverse events were reported. In total, 194 individuals were included in the ITT population and 155 individuals in the CC population. Notably, 39 individuals had missing outcome data at week 16 and were included in the ITT analysis using imputed values.

The oat group consisted of 102 individuals (60% females) and the control group 92 individuals (59% females) (Table 1). The participants included from 4 sites were as follows: Bergen (59 individuals), Gothenburg (42 individuals), Leipzig (40 individuals), and Paderborn (53 individuals). The oat group had a mean HbA1c of 5.6% (SD: 0.3), equivalent to 37.8 mmol/mol (SD: 3.0), whereas the control group had a mean HbA1c of 5.6% (SD: 0.3), equivalent to 38.0 mmol/mol (SD: 3.0). Both groups were normocholesterolemic.

**TABLE 1:** Descriptive and biochemical characteristics of participants at baseline according to group.

Demographic characteristics	Oat (*n* = 102)	Control (*n* = 92)
Age at randomization (y)	57 (8)	58 (9)
Female, *n* (%)	62 (61)	54 (59)
Body weight (kg)	95.5 (16.0)	94.0 (16.2)
BMI (kg/m^2^)	32.3 (4.8)	31.9 (4.4)
Waist circumference (cm)	109 (11)	109 (11)
Metabolic characteristics		
HbA1c (%)	5.6 (0.3)	5.6 (0.3)
HbA1c (mmol/mol)	37.8 (3.0)	38.0 (3.0)
Glucose (mmol/L)	5.6 (0.8)	5.5 (0.6)
Insulin (pmol/L)	13.5 (11.6)	12.3 (8.3)
Total cholesterol (mmol/L)	5.5 (1.0)	5.5 (1.0)
Triglycerides (mmol/L)	1.5 (0.6)	1.4 (0.6)
HDL (mmol/L)	1.5 (0.4)	1.5 (0.4)
LDL (mmol/L)	3.6 (1)	3.6 (1)
Systolic blood pressure (mmHg)	134 (17)	129 (15)
Diastolic blood pressure (mmHg)	85 (9)	83 (10)
Sociodemographic characteristics		
Education level, *n* (%)		
Low	22 (22)	16 (17)
Medium	26 (26)	29 (32)
High	52 (51)	44 (48)
NA	2 (2)	3 (3)
Ethnicity, *n* (%)		
Caucasian	89 (87)	80 (87)
Other	8 (8)	11 (12)
NA	5 (5)	1 (1)
Physical activity, *n* (%)		
Low	20 (20)	34 (37)
Moderate	40 (39)	28 (30)
High	42 (41)	30 (33)

Data are presented as mean ± SD for continuous variables and *n* (%) for categorical variables. Data are based on the ITT population (*n* = 194); missing values at week 16 were imputed using multiple regression imputation.

Abbreviations: HbA1c, hemoglobin A1c; ITT, intention to treat; NA, missing or did not want to reply.

### Dietary intake and intervention bread consumption

At baseline, both the oat and control groups had comparable energy and macronutrient intakes. Over the 16-wk intervention, both groups similarly increased their reported energy intake. Carbohydrate intake decreased similarly in both groups, whereas protein intake increased slightly more in the oat group than in the control group. Fat intake showed a slightly greater increase in the oat group. There was a slight decrease in alcohol intake in both groups. Fiber intake remained similar between the groups by the end of the intervention (Table 2). According to the self-reported journals, most participants in the oat group consumed an average of 3.7 slices per day (SD: 0.7), providing 7.4 g of β-glucans daily over 16 wk. The control group averaged 3.9 slices per day (SD: 1.0), corresponding to 0.08 g of β-glucans. In the oat group, 7.8% failed to meet the target of ≥3 slices per day, compared with 6.5% in the control group. Most participants consumed 3–4 slices (67% oat group and 62% control), whereas 25% (oat) and 29% (control) ate 4–6 slices daily.

**TABLE 2:** Average daily intake of energy and macronutrients estimated from 24-h recalls.

Nutrient	Time	Oat group	Control group
Energy (kcal)	Baseline	1690 (440)	1750 (470)
	Week 16	1820 (470)	1814 (460)
Carbohydrates (E%)	Baseline	37 (7)	37 (6)
	Week 16	32 (7)	33 (6)
Carbohydrates (g/d)	Baseline	155 (49)	162 (49)
	Week 16	145 (49)	148 (49)
Protein (E%)	Baseline	17 (3)	17 (3)
	Week 16	17 (3)	16 (3)
Protein (g/d)	Baseline	72 (19)	71 (17)
	Week 16	77 (22)	72 (15)
Fat (E%)	Baseline	39 (6)	40 (6)
	Week 16	43 (6)	42 (5)
Fat (g/d)	Baseline	74 (24)	78 (25)
	Week 16	87 (27)	85 (24)
Dietary fiber (g/d)	Baseline	20 (7)	21 (7)
	Week 16	21 (9)	22 (10)
Alcohol g/d	Baseline	6 (10)	5 (9)
	Week 16	5 (7)	4 (7)

Data are presented as means and SD. Values expressed as percentages of energy intake (E%) and grams/day. Baseline mean is based on recalls 1 and 2, and week 16 is based on recalls 5 and 6. Data are based on the ITT population (*n* = 194); missing values at week 16 were imputed using multiple regression imputation.

Abbreviation: ITT, intention to treat.

### Metabolic measurements

The primary endpoint, change in HbA1c, did not differ between the oat and the control groups during the intervention (Table 3). Similarly, changes in fasting glucose, fasting insulin, HOMA-IR, and QUICKI between baseline and week 16 did not differ between the oat and the control groups. Ad hoc analysis was performed between the highest and lowest consumer groups (median split) but the number of breads consumed did not alter the results (all *P* values > 0.05).

**TABLE 3:** Clinical measurements at baseline and after 16 wk.

Variable	Timepoint	Oat mean (SD)^1^	Control mean (SD)^1^	Δ (95% CI)^2^	*P* value^3^
HbA1c (%)	Baseline	5.6 (0.3)	5.6 (0.3)	NA	
	16 wk	5.6 (0.2)	5.6 (0.2)	−0.01 (−0.03, 0.06)	0.49
HbA1c (mmol/mol)	Baseline	37.7 (3.0)	38.0 (3.0)	NA	
	16 wk	38.0 (1.7)	38.1 (1.7)	−0.16 (−0.30, 0.63)	0.49
Glucose (mmol/L)	Baseline	5.6 (0.7)	5.5 (0.7)	NA	
	16 wk	5.5 (0.4)	5.5 (0.4)	−0.02 (−0.11, 0.14)	0.77
Insulin (pmol/L)	Baseline	13.7 (10.2)	12.5 (10.1)	NA	
	16 wk	12.5 (6.2)	13.3 (6.2)	−0.76 (−0.99, 2.5)	0.39
HOMA-IR	Baseline	3.6 (3.1)	3.2 (3.1)	NA	
	16 wk	3.2 (1.6)	3.4 (1.6)	−0.26 (−0.20, 0.71)	0.28
QUICKI	Baseline	0.15 (0.02)	0.14 (0.02)	NA	
	16 wk	0.14 (0.01)	0.14 (0.01)	0.00 (−0.00, 0.00)	0.71
Cholesterol (mmol/L)	Baseline	5.5 (1.0)	5.5 (1.0)	NA	
	16 wk	5.5 (0.7)	5.5 (0.7)	0.08 (−0.26, 0.10)	0.39
LDL-C (mmol/L)	Baseline	3.6 (1.0)	3.6 (1.0)	NA	
	16 wk	3.6 (0.6)	3.5 (0.6)	−0.11 (−0.27, 0.05)	0.19
HDL-C (mmol/L)	Baseline	1.5 (0.4)	1.5 (0.4)	NA	
	16 wk	1.5 (0.2)	1.5 (0.2)	−0.00 (−0.05, 0.07)	0.78
Triglycerides (mmol/L)	Baseline	1.5 (0.6)	1.4 (0.6)	NA	
	16 wk	1.4 (0.5)	1.4 (0.5)	0.00 (−0.13, 0.13)	0.98
FLI	Baseline	10.7 (15.3)	10.4 (15.3)	NA	
	16 wk	10.1 (4.7)	10.2 (4.7)	−0.06 (−1.25, 1.37)	0.93
HSI	Baseline	42.6 (5.5)	42.3 (5.5)	NA	
	16 wk	42.4 (2.6)	42.6 (2.6)	−0.17 (−0.55, 0.89)	0.63
CRP^4^ (mg/L)	Baseline	1.7 (1.0)	1.6 (1.0)	NA	
	16 wk	1.9 (0.9)	1.8 (1.0)	0.09 (−0.4, 0.23)	0.58

Oat = 102, control= 92, unless otherwise stated.

Abbreviations: CI, confidence interval; FLI, Fatty Liver Index; HbA1c, hemoglobin A1c; HSI, Hepatic Steatosis Index; NA, not applicable/not available; QUICKI, Quantitative Insulin Sensitivity Check Index.

1 Mean adjusted for study site.

2 Δ between groups represents the difference in adjusted means control—oat between baseline and 16 wk.

3 Generalized linear model. Δ week 16–week 0 as dependent variable, treatment group as factor, week 1 value, and center as included covariates.

4 Values above ≥4.1 mg/L removed. Analyses are based on *n* = 137 (oat = 75, control= 62). Data are based on the ITT population (*n* = 194); missing values at week 16 were imputed using multiple regression imputation.

No change in blood lipid profile between the 2 groups was found (Table 3) (all *P* values > 0.05). Additionally, results were not altered by considering lipid-lowering drug treatment (*n* = 16).

There were no differences in the changes in FLI, steatosis index, or CRP between the groups after the intervention (*P* values > 0.05) (Table 3).

Over the 16-wk intervention, there were no significant differences between the oat and the control groups in terms of changes in body weight, body fat mass, or fat-free mass (Table 4).

**TABLE 4:** Body weight and body composition at baseline and after 16 wk.

Measure (kg)	Timepoint	Oat group∗	Control group∗	Δ (95% CI)^1^	*P* value^2^
Body weight	Baseline	95.4 (16.3)	94.1 (16.3)	NA	
	Week 16	94.3 (4.6)	94.7 (4.6)	0.4 (−0.9, 1.7)	0.55
Body fat mass	Baseline	45.7 (10.1)	45.4 (10.1)	NA	
	Week 16	44.8 (5.5)	44.9 (5.5)	0.08 (−1.5, 0.6)	0.92
Fat-free mass	Baseline	49.5 (10.2)	48.7 (10.2)	NA	
	Week 16	49.3 (4.3)	50.0 (4.3)	0.7 (−0.5, 1.9)	0.23

Data are presented as means ± SD. Mean adjusted for study site.

Abbreviations: CI, confidence interval; ITT, intention to treat; NA, not applicable/not available.

1 Δ between groups represents the difference in adjusted means control—oat between baseline and 16 wk.

2 Generalized linear model. Δ week 16–week 0 as dependent variable, treatment group as factor, week 1 value, and center as included covariates. Data are based on the ITT population (*n* = 194); missing values at week 16 were imputed using multiple regression imputation.

### The intervention breads and their acceptance, and effects self-reported appetite

Overall, the bread types were equally accepted both at the start of the trial and at week 8. However, there were some differences in acceptance between the countries. German subjects had a significantly greater consumer acceptance for the oat bread at week 8 (mean ± SD: 5.9 ± 1.1) than Norwegians (3.7 ± 1.8). Participants reported significantly overall greater satiety (4.6 ± 0.8) and satiation (6.9 ± 1.7) after the intake of oat bread than after the intake of control bread (satiety: 4.3 ± 1.05; satiation: 6.2 ± 1.69) There was no difference between satiety and satiation between day 1 and week 8, or between countries (Supplemental Tables 6 and 7).

The participants’ description of the breads is presented in Supplemental Table 8, showing answers to the check-all-that-apply question (sensory and nonsensory attributes) they considered applied to the consumed sample (only significant attributes among samples and evaluation weeks are presented).

## Discussion

The CarbHealth trial is, to our knowledge, the first study to evaluate the effectiveness of habitual consumption of an oat β-glucan–enriched bread on cardiometabolic risk markers among individuals with elevated type 2 diabetes risk. Contrary to findings from controlled efficacy studies, no significant improvements in HbA1c or other glycemic control parameters were observed compared with a whole-grain control bread. This suggests that the real-world effectiveness of the intervention may differ from the effects seen under controlled conditions.

Several studies have demonstrated beneficial effects of β-glucans on glycemic control, under controlled conditions [30], but these may not extend to real-world settings. Our findings align with previous effectiveness studies showing a gap between controlled trial efficacy and real-world outcomes [31].

We previously evaluated the acute postprandial glucose and insulin responses of the intervention breads used in the present trial among 22 healthy adults under carefully controlled conditions [32] and found a substantial reduction in postprandial glucose (incremental area under the curve_2h_) for the oat bread [38.9 (22.9, 66.1) mmol/L ∗ min] compared with the control bread [87.1 (65.4, 115.9) mmol/L ∗ min] [32]. Although these results suggest short-term benefits in healthy individuals, the observed effects did not translate into meaningful long-term improvements in glycemic control under real-world conditions in this study.

According to an EFSA- health claim, 4 g of β-glucans consumed per meal could reduce postprandial blood glucose [10]. Although the oat bread met EFSA’s claim, our flexible design likely led to inconsistent intake of the recommended 4 g per meal. This suggests that simple substitution of the bread, without guidance on timing and quantity, may not be sufficient to achieve the desired health effects.

Adherence and proper implementation are crucial factors in determining the success of effectiveness trials [31,33]. In our study, adherence to the protocol was estimated through self-reported journals. Weak adherence or acceptance of the intervention might explain the lack of significant changes observed in our trial. For example, adherence rates in similar dietary interventions, like the Prevención con Dieta Mediterránea (PREDIMED) trial, varied between 54% and 58%, with factors like female sex, cardiovascular disease risk, obesity, and type 2 diabetes linked to lower compliance. [34]. Good bread acceptance likely supported compliance, as the oat bread was perceived as more satiating compared with the control, with sensory attributes such as being more compact, doughy, heavy, juicy, sticky, and less dry—characteristics previously linked to increased satiation [35].

Overall, these findings illustrate the challenge of translating controlled efficacy results to real-world settings, where adherence, individual variability, and diet context affect outcomes. Future research should explore consumption patterns and barriers to improve real-world effectiveness of dietary interventions

One potential explanation for the lack of effectiveness in our study is the relatively healthy metabolic profile of the participants, who had a mean baseline HbA1c of 5.6% (∼38.0 mmol/mol). Meta-analyses have plausibly shown that β-glucans tend to have more pronounced effects on glycemic control in individuals with type 2 diabetes or those with higher baseline glycemic levels, whereas individuals without established diabetes may not experience similar benefits [30,36].

Beyond aspects relating the design of an effectiveness trial, the lack of effectiveness on fasting insulin, glucose levels, or insulin indices also aligns with results from a meta-analysis by Bao et al. [36], which reported no significant improvements in fasting glucose concentrations or HOMA-IR in 13 controlled efficacy studies with 3–10 g β-glucans per day for 1–8 wk. He et al. [30] highlighted substantial heterogeneity across studies, suggesting that the effects of β-glucans differ depending on factors such as study duration, health status, study design, amount, and molecular weight of β-glucans.

The lack of improvement in blood lipid profiles in this study was expected because participants were in general normocholesterolemic. The LDL-lowering effect of β-glucan depends on elevated baseline values [8].

### Strengths and limitations

Strengths of this study include its multicenter design with a large sample size, covering different countries in Europe that have distinct bread consumption cultures. This allowed us to compare the metabolic response and effectiveness of the bread across diverse bread consumption patterns and habitual diets. Additional strengths include strong design features such as blinding, randomization, provision of bread, and a long intervention period.

Moreover, the choice of a whole-grain wheat bread as a comparator is another strength of our study. A review by Tosh [7] emphasized the importance of a suitable control to provide a more precise estimate of the effects of β-glucans on postprandial glucose response. The selection of whole-grain wheat bread as a comparator aligns well with everyday dietary habits in Sweden, Norway, and Germany, where whole-grain and mixed-grain breads are commonly consumed [37]. The breads were matched for starch and fat content, and the oat bread contained a high concentration of high-molecular-weight β-glucans. Additionally, the oat bread’s glycemic response was evaluated separately among healthy participants, further strengthening the study’s design [32].

One limitation of the study is that, due to logistics, the breads had to be provided frozen, which is known to reduce bread quality. Frozen storage of oat bread is known to lower β-glucan solubility and molecular weight [38,39]. However, in vitro digestion of the intervention bread following its 2-y frozen storage still showed that the β-glucan molecular weight and solubility remained above the critical coil overlap threshold. [40,41].

We acknowledge that we did not make a thorough assessment of the habitual diet of the participants at study entry, which makes it difficult to rule out that there may have been differences in the habitual diet that may have interacted with the intervention. Furthermore, although the intervention and control breads differed substantially in fiber content, we observed no significant between-group difference in reported dietary fiber intake at week 16. This may reflect compensation from other fiber sources in the habitual diet, which we did not capture in detail. However, it should be noted that according to EFSA guidelines, background diet should not be considered when evaluating the efficacy of a specific dietary component; the effect must be demonstrated under the proposed conditions of use within normal dietary patterns [42]. In this context, our pragmatic trial design, with participants from 3 different countries where bread is a major staple food, can be considered a strength. Moreover, we cannot rule out lack of compliance because a 6-time repeated 24-recalls along with tick-off lists where participants recorded their consumption of the intervention foods every day, may not fully capture their compliance. Recently developed methods for analysis of avenanthramides and avenacosides, putative biomarkers of oat intake under intervention conditions, may allow independent assessment of compliance in the future. Another limitation is the lack of detailed data on bread consumption context (for example, with meals), which could inform how the bread was integrated into participants’ diets and whether the intended β-glucan dose was achieved.

Additionally, although the study did not reach its recruitment goal of 250 participants, this could be raised as a potential limitation affecting the detection of significant effects. However, the dropout rate was lower than anticipated, which helped maintain a sufficient sample size to meet the power calculation requirements.

Finally, most participants were of Caucasian ethnicity, limiting the generalizability of findings to more diverse populations.

The CarbHealth trial, a large-scale dietary intervention, found no significant improvements in glycemic control after 16 wk of consuming β-glucan–enriched bread that provided ≥6 g β-glucan per day in individuals at elevated risk of developing type 2 diabetes. This suggests that replacing a healthy control whole-grain bread with β-glucan–enriched alternative may not be enough to improve glycemic control in these individuals. Further research is needed to determine under which conditions β-glucans are effective in real-life settings with the potential to benefit long-term metabolic health outcomes.

## Author contributions

The author responsibilities were as follows: TH: wrote the first draft of the manuscript; JD: main responsible investigator of CarbHealth consortium; HR-R: responsible investigator for the multicenter trial; RL, AEB, AK: responsible for the respective sites; SB: responsible for developing and characterization of the breads; PV: responsible for the consumer acceptance of the study bread; JD, RL, AEB, AK, HR-R, SB: research question, study design, acquisition of data, obtaining the funding, implementation of the study protocol, critical revision; TH: statistical analyses, first interpretation of results; and all authors: interpretation of results, implementation of study protocol, acquisition of data, critical revision, and final approval of the manuscript.

## Data availability

The data presented in this study are available on reasonable request from the corresponding author.

## Funding

This project has received funding from the Research Council of Norway, Formas Research Council of Sweden, the Federal Ministry of Education and Research (Germany) under the umbrella of the European Joint Programming Initiative “A Healthy Diet for a Healthy Life” (JPI HDHL) and of the ERA-NET Cofund HDHL INTIMIC (grant no. 727565) of the EU Horizon 2020 Research and Innovation Programme (grant number N/A). The funding agencies did not play any part in designing the clinical trial or reporting the results.

## Conflict of interest

AS, US, KM, AK, and AEB report financial support from the Federal Ministry of Education and Research, Bonn Office. IR, AR, PV, SB, JD, and HR-R report financial support from the Research Council of Norway. AR and SB report provision of equipment, drugs, or supplies from Lantmännen Cerealia AB. RL recieves financial support from the Swedish Research Council Formas and discloses relationships with Lantmännen Research Foundation and Arla Food and Health, including consulting or advisory roles; RL is the project leader of several projects funded by Lantmännen Research Foundation, Oatly, and Barilla; and serves as a scientific evaluator for both Lantmännen Research Foundation and Arla Food and Health. SB further reports a relationship with Lantmännen Research Foundation that includes funding grants. AEB is a member of the International Carbohydrate Quality Consortium, the Scientific Committee for the transnational governance of the Nutri-Score system, and the Scientific Advisory Council for Agricultural Policy, Nutrition, and Consumer Health Protection under the Federal Ministry of Food and Agriculture, Germany; also a coauthor of the popular cookbook Nordisch abnehmen; RL is the founder of the Nordic Rye Forum, a collaboration platform between industry, academia, and institutes in the Nordic countries with an interest in rye and health; and also serves as an Associate Editor of Nutrition. The Rye Forum is funded by membership fees from industrial partners.
