# Supplementary material for: Effectiveness of regular oat β-glucan–enriched bread compared with whole-grain wheat bread on long-term glycemic control in adults at risk of type 2 diabetes: a randomized controlled trial
Source: Am J Clin Nutr. 2025 Jun 24;122(3):724–32. doi: 10.1016/j.ajcnut.2025.06.018 (PMC12489378; doi:10.1016/j.ajcnut.2025.06.018)
Supplement: multimedia component 1 [file mmc1.docx]

**Effectiveness of Regular Oat β-Glucan Enriched Bread Consumption Compared to Whole Grain Wheat Bread on Long-Term Glycemic Control in Adults at Risk of Type 2 Diabetes: Results from the CarbHealth Randomized Controlled Trial**

**Authors:** Therese Hjorth

**Supplementary Table S1.** Descriptive and biochemical characteristics of participants at baseline according to group for the complete case analysis (Oat = 78, Control = 77).

| Demographic Characteristics | Oat | Control |
| --- | --- | --- |
| Age at randomization (years) | 58.0 (8) | 60 (8) |
| Female *n* (%) | 47 (60) | 47 (61) |
| Body Weight (kg) | 95.9 (17.7) | 92.5 (12.4) |
| BMI (kg/m^2^) | 32.4 (4.9) | 31.7 (3.8) |
| Waist Circumference (cm) | 109.0 (11.5) | 108.0 (9.8) |
| Metabolic Characteristics | | |
| Hba1c % | 5.6 (0.3) | 5.6 (0.3) |
| Hba1c (mmol/mol) | 37.7 (3.0) | 38.2 (3.2) |
| Glucose (mmol/L) | 5.6 (0.8) | 5.5 (0.6) |
| Insulin (pmol/L) | 13.7 (12.3) | 12.3 (8.2) |
| Total cholesterol (mmol/L) | 5.7 (1.0) | 5.6 (1.0) |
| Triglycerides (mmol/L) | 1.5 (0.7) | 1.5 (0.7) |
| HDL (mmol/L) | 1.5 (0.4) | 1.5 (0.4) |
| LDL (mmol/L) | 3.7 (1.0) | 3.6 (0.9) |
| Systolic blood pressure (mm Hg) | 135.0 (16.9) | 130.0 (14.0) |
| Diastolic blood pressure (mm Hg) | 85.2 (8.6) | 83.3 (9.0) |
| Sociodemographic Characteristics | | |
| Education level *n* (%) |  |  |
| Low | 18 (34%) | 14 (19%) |
| Medium | 22 (29%) | 25 (34%) |
| High | 37 (48%) | 35 (47%) |
| Ethnicity, *n* (%) | 91/8/1 | 88/10/1 |
| Caucasian | 68 (91%) | 68 (88%) |
| Other | 6 (8%) | 8 (10%) |
| NA | 1 (1%) | 1 (1%) |
| Physical activity, *n* (%) |  |  |
| Low | 15 (19%) | 24 (31%) |
| Moderate | 26 (33%) | 26 (34%) |
| High | 37 (47%) | 27 (35%) |

Data are presented as mean ± SD for continuous variables and n (%) for categorical variables. NA = Missing or did not want to reply. BMI, Body Mass Index; HbA1c, hemoglobin A1c; HDL, high-density lipoprotein; LDL, low-density lipoprotein.

**Supplementary Table S2.** Average daily intake of energy and macronutrients estimated from 24-hour recalls for the complete case analysis (Oat = 78, Control = 77).

| Nutrient | Time | Oat | Control |
| --- | --- | --- | --- |
| Energy (kcal) | Baseline | 1684 (552) | 1778 (605) |
|  | Week 16 | 1845 (680) | 1818 (589) |
| Carbohydrates (E%) | Baseline | 35.2 (10.7) | 36.6 (9.0) |
|  | Week 16 | 41 (33) | 34 (15) |
| Carbohydrates (g/d) | Baseline | 146 (62) | 163 (63) |
|  | Week 16 | 149 (72) | 146 (58) |
| Protein (E%) | Baseline | 18.0 (4.7) | 16.7 (4.3) |
|  | Week 16 | 17.8 (5.0) | 16.0 (3.6) |
| Protein (g/d) | Baseline | 73.3 (25.5) | 72.2 (25.3) |
|  | Week 16 | 79.8 (31.2) | 70.2 (19.8) |
| Fat (E%) | Baseline | 39 (11) | 39 (11) |
|  | Week 16 | 50.4 (32) | 45 (21) |
| Fat (g/d) | Baseline | 74 (32) | 79 (37) |
|  | Week 16 | 87 (38) | 86 (34) |
| Dietary fiber (g/d) | Baseline | 19 (9) | 21 (9) |
|  | Week 16 | 22 (14) | 23 (13) |
| Alcohol g/d | Baseline | 6 (13) | 5 (13) |
|  | Week 16 | 4 (9) | 4 (8) |

Data presented as Means and SD. Values expressed as percentages of energy intake (E%) and gram/day. Baseline mean is based on recalls 1 and 2, Week 16 is based on recalls 5 and 6.

**Supplementary Table S3.** Clinical measurements at baseline and after 16 weeks for the complete case analysis. (Oat = 78, Control= 77, unless otherwise stated).

| Variable | Timepoint | Oat mean (SD)* | Control mean (SD)* | Δ (95% CI)^†^ | P-value^‡^ |
| --- | --- | --- | --- | --- | --- |
| HbA1c (%) | Baseline | 5.6 (0.3) | 5.6 (0.3) |  |  |
|  | 16 weeks | 5.6 (0.2) | 5.6 (0.2) | 0.01 (-0.04, 0.06) | 0.62 |
| HbA1c (mmol/mol) | Baseline | 37.6 (3.1) | 38.0 (3.1) |  |  |
|  | 16 weeks | 37.9 (1.6) | 38.0 (1.7) | 0.13 (-0.38, 0.64) | 0.62 |
| Glucose (mmol/L) | Baseline | 5.6 (0.7) | 5.5 (0.7) |  |  |
|  | 16 weeks | 5.5 (0.4) | 5.5 (0.4) | -0.00 (-0.14, 0.13) | 0.93 |
| Insulin (pmol/L) | Baseline | 13.9 (10.4) | 12.7 (10.5) |  |  |
|  | 16 weeks | 12.5 (6.5) | 13.0 (6.5) | 0.42 (-1.62, 2.45) | 0.69 |
| HOMA-IR | Baseline | 3.7 (3.4) | 3.3 (3.4) |  |  |
|  | 16 weeks | 3.2 (1.7) | 3.3 (1.7) | 0.15 (-0.39, 0.69) | 0.58 |
| QUICKI | Baseline | 0.15 (0.02) | 0.14 (0.02) |  |  |
|  | 16 weeks | 0.14 (0.01) | 0.14 (0.01) | -0.00 (-0.00, 0.00) | 0.86 |
| Cholesterol (mmol/L) | Baseline | 5.6 (1.0) | 5.5 (1.0) |  |  |
|  | 16 weeks | 5.5 (0.6) | 5.5 (0.6) | -0.01 (-0.20, 0.17) | 0.90 |
| LDL-C (mmol/L) | Baseline | 3.7 (1.0) | 3.6 (1.0) |  |  |
|  | 16 weeks | 3.6 (0.6) | 3.5 (0.6) | -0.05 (-0.22, 0.13) | 0.60 |
| HDL- C (mmol/L) | Baseline | 1.5 (0.4) | 1.5 (0.4) |  |  |
|  | 16 weeks | 1.5 (0.2) | 1.5 (0.2) | 0.00 (-0.05, 0.07) | 0.79 |
| Triglycerides (mmol/L) | Baseline | 1.5 (0.7) | 1.5 (0.7) |  |  |
|  | 16 weeks | 1.4 (0.5) | 1.4 (0.5) | -0.03 (-0.19, 0.12) | 0.66 |
| FLI | Baseline | 11.3 (13.9) | 9.1 (14.0) |  |  |
|  | 16 weeks | 9.5 (4.4) | 9.7 (4.4) | 0.23 (-1.13, 1.60) | 0.74 |
| HSI | Baseline | 43.0 (5.4) | 42.2 (5.5) |  |  |
|  | 16 weeks | 42.2 (2.2) | 42.4 (2.3) | 0.16 (-0.54, 0.86) | 0.65 |
| CRP^§^ (mg/L) | Baseline | 1.6 (1.0) | 1.6 (1.1) |  |  |
|  | 16 weeks | 1.5 (0.9) | 1.7 (1.0) | 0.18 (-0.14, 0.50) | 0.27 |

HbA1c, hemoglobin A1c; HOMA-IR, homeostasis model assessment insulin resistance; QUICKI, quantitative insulin sensitivity check index; LDL-C, low density lipoprotein cholesterol; HDL-C, high density lipoprotein cholesterol, FLI fatty liver index, HSI hepatic steatosis index. * Mean adjusted for study cite, ^†^ Δ between groups represents the difference in adjusted means control - oat between baseline and 16 weeks, ^‡^ Generalized linear model. Δ week 16 – week 0 as dependent variable, treatment group as factor, week 1 value and center as included covariates, ^§^Values above ≥ 4.1 mg/L removed. Analyses are based on n = 111 (Oat = 56, Control= 55).

**Supplementary Table S4.** Body weight and body composition at baseline and after 16 weeks for the complete case analysis (Oat = 78, Control = 77).

| Measure (kg) | Timepoint | Oat group* | Control group* | Δ (95% CI)^†^ | P-value^‡^ |
| --- | --- | --- | --- | --- | --- |
| Body weight | Baseline | 95.9 (19.0) | 92.6 (15.6) |  |  |
|  | Week 16 | 93.6 (2.7) | 94.0 (2.2) | 0.4 (-0.3, 1.1) | 0.26 |
| Body fat mass | Baseline | 45.9 (12.4) | 44.0 (12.4) |  |  |
|  | Week 16 | 44.1 (3.4) | 44.2 (3.4) | 0.1 (-0.8, 1.1) | 0.79 |
| Fat free mass | Baseline | 49.9 (12.4) | 48.6 (12.5) |  |  |
|  | Week 16 | 49.2 (3.6) | 49.5 (3.6) | 0.3 (-0.7, 1.3) | 0.52 |

Data is presented as means ± SD. ^*^ Mean adjusted for study site, ^†^ Δ between groups represents the difference in adjusted means control- oat between baseline and 16 weeks, ^‡^ Generalized linear model. Δ week 16 – week 0 as dependent variable, treatment group as factor, week 1 value and center as included covariates.

**Supplementary Table S5.** Average number of slices of bread consumed per day for the complete case analysis (Oat = 78, Control = 77).

| Group | Average/day* | β-glucans^†^ | ≥ 3 slices^‡^ | 3-4 slices^§^ | 4-6 slices^\|\|^ | < 3 slices^¶^ |
| --- | --- | --- | --- | --- | --- | --- |
| Oat | 3.5 (0.8) | ~ 6g | 92.4% | 80.9% | 10.1% | 7.6% |
| Control | 3.6 (1.0) | *NA* | 95.0 | 78.9% | 13.6% | 5% |

^*^ Mean number of slices consumed per day (SD), ^†^ Mean daily intake of β-glucans, ^‡^ Percentage of participants consuming ≥ 3 slices/day, ^§^ Percentage of participants consuming 3-4 slices/day, ^||^ Percentage of participants consuming 4-6 slices/day, ^¶^ Percentage of participants consuming < 3 slices/day.

**Supplementary Table S6**. Mean scores for acceptance, satiety, and satiation of the intervention breads at baseline and week 8 by country and treatment group (oat vs. control).

| Timepoint | Variable | Norway Oat | | Norway Control | | Sweden Oat | | Sweden Control | | Germany Oat | | Germany Control | |
| --- | --- | --- | --- | --- | --- | --- | --- | --- | --- | --- | --- | --- | --- |
|  |  | n | Mean (± SD |  | |  | |  | |  | |  | |
| Baseline | Acceptance | 21 | 4.8 (1.7)^AB^ | 20 | 5.2 (1.4)^AB^ | 18 | 4.9 (1.0)^AB^ | 19 | 5.2 (1.7)^AB^ | 12 | 5.8 (0.8)^AB^ | 10 | 5.1 (2.3)^AB^ |
| Baseline | Satiety | 21 | 4.3 (1.0)^A^ | 20 | 4.5 (0.9)^A^ | 18 | 4.4 (0.6)^A^ | 19 | 4.0 (0.2)^A^ | 12 | 4.9 (0.8)^A^ | 9 | 4.0 (1.1)^A^ |
| Baseline | Satiation | 21 | 6.1 (1.9)^A^ | 20 | 6.0 (1.6)^A^ | 18 | 6.9 (1.2)^A^ | 19 | 6.5 (1.8)^A^ | 12 | 7.1 (1.8)^A^ | 10 | 5.8 (1.9)^A^ |
| Week 8 | Acceptance | 15 | 3.7 (1.8)^B^ | 16 | 5.3 (1.3)^AB^ | 16 | 4.3 (1.5)^AB^ | 19 | 4.9 (2.4)^AB^ | 14 | 5.9 (1.0)^A^ | 12 | 5.1 (2.2)^AB^ |
| Week 8 | Satiety | 15 | 4.7 (0.8)^A^ | 16 | 4.4 (1.3)^A^ | 16 | 4.3 (1.1)^A^ | 19 | 4.3 (1.1)^A^ | 14 | 5.1 (0.7)^A^ | 12 | 4.3 (1.0)^A^ |
| Week 8 | Satiation | 16 | 7.1 (1.8)^A^ | 16 | 6.2 (1.9)^A^ | 16 | 6.4 (1.8)^A^ | 19 | 6.4 (1.7)^A^ | 14 | 7.9 (1.3)^A^ | 12 | 6.3 (1.4)^A^ |

Data presented as mean ± SD. Means that do not share a letter for each variable are significantly different. Data based on CC population (oat n = 78, control n = 77).

**Supplementary Table S7**. Mean scores for satiety and satiation of the intervention breads (oat vs. control).

| Bread | Variable | N | Mean (± SD) |
| --- | --- | --- | --- |
| Oat | Satiety | 96 | 4.6 (0.8)^A^ |
| Control |  | 96 | 4.3 (1.0)^B^ |
|  |  |  |  |
| Oat | Satiation | 96 | 6.9 (1.7)^A^ |
| Control |  | 96 | 6.2 (1.7)^B^ |

Data presented as mean ± SD. Means that do not share a letter for each variable are significantly different. Data based on CC population (oat n = 78, control n = 77).

**Supplementary Table S8.** Consumers description of the breads by answering the Check-all that-Apply question (n=117). Results show the frequency of selection of the attributes that were significantly different in the Cochran and McNemar tests (different letters mean significant differences at 5%).

| Attribute/ Product | Oat, week 1 | Oat, week 8 | Wheat, week 1 | Wheat, week 8 |
| --- | --- | --- | --- | --- |
| Acidic taste | 4^AB^ | 0^BC^ | 0^C^ | 4^A^ |
| Raw taste | 9^AB^ | 11^A^ | 3^BC^ | 2^C^ |
| Cloying taste | 0^B^ | 2^AB^ | 4^A^ | 3^AB^ |
| Compact | 34^A^ | 32^A^ | 19^B^ | 25^AB^ |
| Fibrous | 4^AB^ | 3^B^ | 6^AB^ | 10^A^ |
| Doughy | 32^A^ | 29^A^ | 7^B^ | 7^B^ |
| Dry | 9^B^ | 5^B^ | 32^A^ | 26^A^ |
| Heavy | 23^A^ | 16^A^ | 4^B^ | 5^B^ |
| Juicy | 14^A^ | 5^B^ | 4^B^ | 6^AB^ |
| Sticky | 14^B^ | 23^A^ | 3^C^ | 8^BC^ |
| Coarse | 1^B^ | 2^AB^ | 4^AB^ | 7^A^ |
| Airy | 3^AB^ | 0^B^ | 2^AB^ | 4^A^ |
| Filling | 32^AB^ | 32^A^ | 24^B^ | 25^AB^ |
| Light | 3^B^ | 3^B^ | 10^A^ | 11^A^ |
| For packed lunch | 23^A^ | 13^AB^ | 19^AB^ | 12^B^ |
| "Every day" bread | 25^AB^ | 17^B^ | 23^AB^ | 30^A^ |
| If the price was same as the bread I regularly buy, I would buy it | 9^AB^ | 5^B^ | 10^AB^ | 13^A^ |
